# BaGGLS: a Bayesian shrinkage framework for interpretable modeling of interactions in high-dimensional biological data

**DOI:** 10.1093/bioinformatics/btag441

**Published:** 2026-08-21

**Authors:** Marta S Lemanczyk, Lucas Kock, Johanna Schlimme, Nadja Klein, Bernhard Y Renard

**Affiliations:** Hasso Plattner Institute, Digital Engineering Faculty, University of Potsdam, Potsdam, Germany; Department of Statistics and Data Science, National University of Singapore, Singapore, Singapore; Hasso Plattner Institute, Digital Engineering Faculty, University of Potsdam, Potsdam, Germany; Scientific Computing Center, Karlsruhe Institute of Technology, Karlsruhe, Germany; Hasso Plattner Institute, Digital Engineering Faculty, University of Potsdam, Potsdam, Germany

## Abstract

**Motivation:**

Biological data is often high dimensional, noisy, and governed by complex interactions among sparse signals. This poses major challenges for interpretability and reliable feature selection. Tasks such as identifying motif interactions in genomics exemplify these difficulties, as only a small subset of biologically relevant features (e.g. motifs) are typically active, and their effects are often non-linear and context-dependent. While statistical approaches often result in more interpretable models, deep learning models have proven effective in modeling complex interactions and prediction accuracy, yet their black-box nature limits interpretability.

**Results:**

We introduce BaGGLS, a flexible and interpretable probabilistic binary regression model designed for high-dimensional biological inference involving feature interactions. BaGGLS incorporates a Bayesian group global-local shrinkage prior, aligned with the group structure introduced by interaction terms. This prior encourages sparsity while retaining interpretability, helping to isolate meaningful signals and suppress noise. To enable scalable inference, we employ a partially factorized variational approximation that captures posterior skewness and supports efficient learning even in large feature spaces. In extensive simulations, we compare BaGGLS to frequentist probit regressions (unconstrained and with L1-penalty) as well as a probit model with Markov Chain Monte Carlo (MCMC) sampling under a horseshoe prior. We can show that BaGGLS outperforms the other methods with regard to interaction detection and is many times faster than MCMC sampling under the horseshoe prior. We also demonstrate the usefulness of BaGGLS in the context of interaction discovery from motif scanner outputs (e.g. Find Individual Motif Occurrences (FIMO)) and noisy attribution scores from deep learning models. This shows that BaGGLS is a promising approach for uncovering biologically relevant interaction patterns, with potential applicability across a range of high-dimensional tasks in computational biology.

**Availability:**

Code is available at gitlab.com/dacs-hpi/baggls.

## 1 Introduction

One of the greatest methodological challenges in the biomedical data domain is feature selection in high-dimensional data (Yang *et al*. 2021, Borah *et al*. 2024). This challenge has historically been central in genomics, but with deep learning (DL) becoming the dominant analytical paradigm and post hoc attribution being routinely used for interpretability, the problem manifests in a different and more intricate form today. DL models excel at learning complex patterns in genomic sequences (Ismail *et al*. 2025), However, the resulting models are highly nonlinear and operate in settings where biological features are both strongly interactive (Forsberg *et al*. 2017, Xie *et al*. 2025) and extremely sparse (Wheeler *et al*. 2016). These properties amplify the already difficult task of interpreting which features matter and, crucially, how they interact.

As a result, we continue to struggle to reliably explain feature interactions (Borah *et al*. 2024). This difficulty is not merely algorithmic, but rooted in statistical challenges. Among these challenges are the curse of dimensionality, the tendency of interactions to explode combinatorically, sparsity of true effects, and high noise levels (Giraud 2021). These challenges are particularly pronounced when working with post hoc attribution maps. Attribution scores are often noisy and spurious (Majdandzic *et al*. 2023), and aggregating them to motif-level features produces high-dimensional, sparsely informative predictors whose reliability and error are difficult to determine. Altogether, this creates a setting in which classical feature selection approaches fall short, and even modern statistical approaches do not resolve the core problem reliably.

Consequently, we see a strong need for new methodology that addresses the specific structure of post hoc attribution in genomic deep learning. Here, we propose a structured binary regression model that incorporates a large number of potential main effects and interaction terms into its linear predictor. Depending on the application, several potential effect types—such as minimum/maximum operations, logical relationships, and higher-order interactions—can be specified *a priori*; we focus on strict pairwise co-occurrence interactions in our empirical work. For interpretability and reliable identification of relevant effects, we impose sparsity on the coefficients. Within the Bayesian framework, sparsity is naturally enforced through shrinkage priors (George *et al*. 1997, Ishwaran *et al*. 2005, Liang *et al*. 2008, Yanchenko *et al*. 2025). Many continuous shrinkage priors such as the Bayesian Lasso (Park and Casella 2008) and the horseshoe (Carvalho *et al*. 2010) follow a global–local structure (Polson and Scott 2011), in which global parameters jointly shrink coefficients toward zero while local parameters allow coefficient-specific deviations. Xu *et al*. (2017) extend this framework to include group based shrinkage parameters, facilitating structured regularization when coefficients can be meaningfully organized into groups. The inclusion of interaction terms results in many overlapping groups, where each group is formed of all terms involving a specific feature. To accommodate this structure, we extend the group-based shrinkage framework of Xu *et al*. (2017), adapting it to handle the extremely large number of potential interaction terms present in genomic data. A detailed description of the resulting prior structure is given in Section 2.2.

Exact Bayesian inference in the resulting high-dimensional Bayesian probit model is challenging and we propose a computationally efficient variational inference (VI; Blei *et al*. 2017) algorithm, a commonly applied technique in high-dimensional binary regression models (Zhang *et al*. 2019, Ray *et al*. 2020). Here, we extend the approach of Fasano *et al*. (2022) to our novel prior and consider a unified skew–normal (Arrelano-Valle *et al*. 2006) approximation for the regression coefficients. This allows more flexibility than the commonly employed mean field approximation (e.g. Durante *et al*. 2019). The unified skew-normal distribution generalizes the Gaussian distribution to include skewness. Recently, skewness perturbed variational approximations were considered by several authors (e.g. Tan and Chen 2025, Kock *et al*. 2026, Pozza *et al*. 2026) theoretically justified by the skewed Bernstein-von Mises theorem (Durante *et al*. 2024). The unified skew-normal distribution is also a conjugate prior to the probit model (Durante *et al*. 2019, Anceschi *et al*. 2023), so that the variational approximation can be efficiently learned using an analytic coordinate ascent updating scheme (e.g. Bishop 2006, Ray and Szabó 2022). This enables scalable inference even when the number of interactions is large.

Here, we introduce our method *Bayesian Group Global Local Shrinkage* (BaGGLS) and demonstrate that it outperforms state-of-the-art methods in both computational efficiency and interpretability. Importantly, we also show empirically that BaGGLS fills a methodological gap in post hoc analysis of genomic deep learning models. Many post hoc approaches attempt to understand learned regulatory mechanisms by detecting motifs through attribution scores (Bartoszewicz *et al*. 2021, Novakovsky *et al*. 2023). Attribution methods assign position-wise scores to sequences, and downstream tools such as TF-MoDISco (Shrikumar *et al*. 2018) extract motifs from these maps. However, these workflows do not capture interactions between motif patterns, even though such interactions are central to genomic mechanisms (Xie *et al*. 2025).

Motif scanners such as FIMO (Grant *et al*. 2011) overcome some limitations by identifying matches to known PWMs from databases like JASPAR (Rauluseviciute *et al*. 2024). Yet this produces a high-dimensional feature space containing hundreds of motifs, many of which produce spurious matches. When combined with noisy attribution scores, only a small subset of motifs, and often their interaction, contribute meaningfully to phenotypes. These conditions create exactly the type of high-dimensional, sparse, interaction-rich scenario where classical methods struggle. We show that BaGGLS successfully extracts these interacting signals and provides reliable interpretability.

## 2 Bayesian group global-local shrinkage

### 2.1 Model formulation

We consider the probit model


(1) $$y_{i}|\beta ∼\mathrm{Bern}(\Phi (x_{i}^{⊤}\beta ))\;i=1,\ldots n$$

Where Bern(π) denotes the Bernoulli distribution with success probability π, Φ is the cumulative distribution function of the standard normal distribution, $x_{i}={\left(x_{i1},\ldots ,x_{ip}\right)}^{⊤}$ is a vector of pre-defined effects including an intercept and interactions, and $\beta \in R^{p}$ is a vector of model parameters to be learned. By introduction of latent variables $z={\left(z_{1},\ldots ,z_{n}\right)}^{⊤}$ we can augment (1) as


$$y_{i}=I(z_{i}>0),\;z_{i}|\beta ∼N(x_{i}^{⊤}\beta ,1),$$

**Figure 1:**
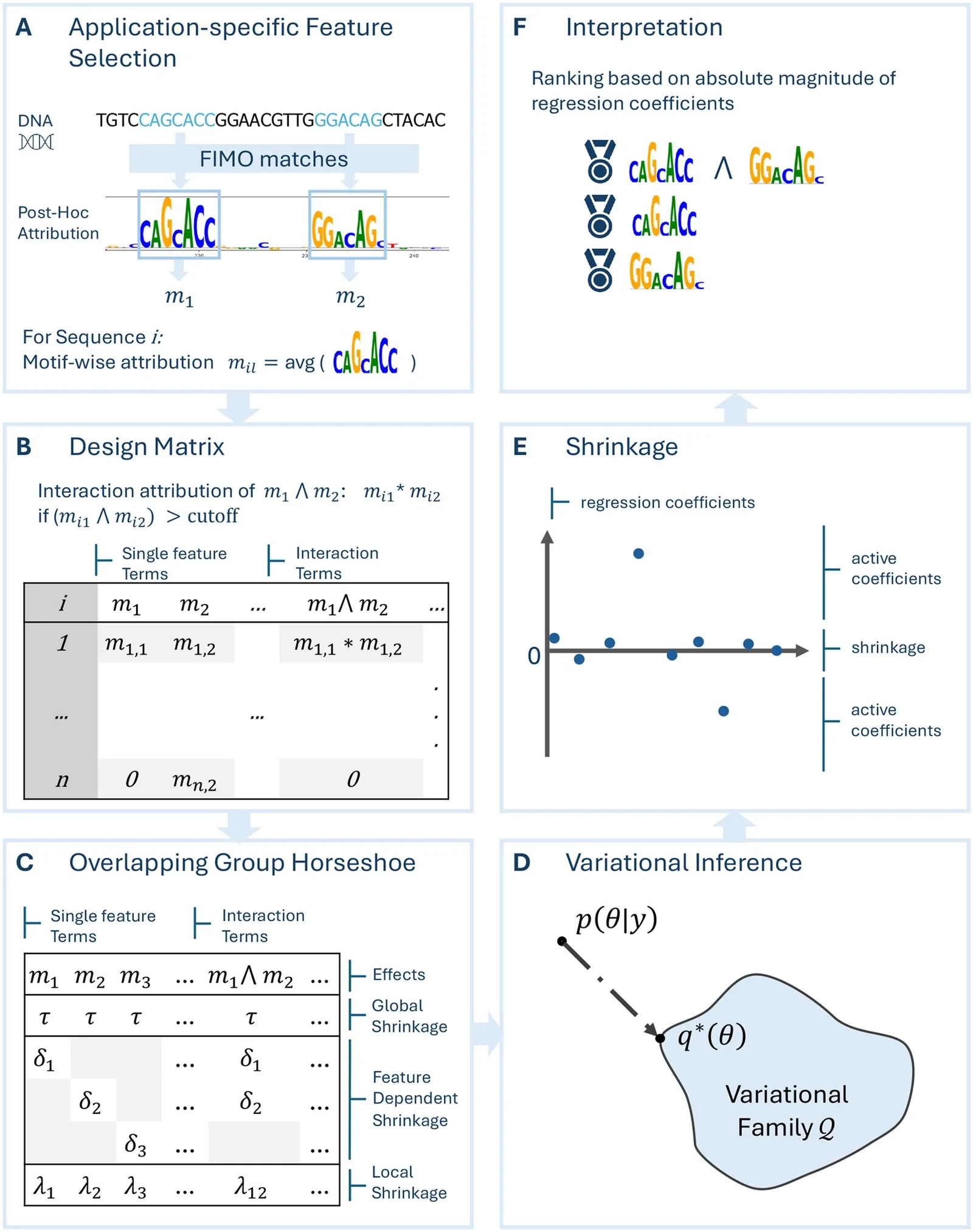
Schematic description of the BaGGLS-workflow. (A) Feature selection based on the observed data. (B) Based on expert knowledge a pre-defined set of potential effects including interpretable interactions is defined. (C) Our novel overlapping group horseshoe prior matches the structure of the pre-defined effect terms and allows for global, local, and feature dependent shrinkage of the regression coefficients. (D) Scalable Bayesian inference is carried our by projecting the true posterior onto a tractable family of variational posteriors Q. (E) The prior shrinks most regression coefficients towards 0 resulting in a sparse and interpretable regression model. (F) For interpretation, we rank the effects based on absolute magnitude of their corresponding coefficients in the regression model.

Where $N(\mu ,\sigma^{2})$ denotes a normal distribution with mean μ and variance $\sigma^{2}$, and $I(z_{i}>0)$ denotes the indicator function that takes a value of 1 if $z_{i}>0$ and 0 otherwise. This representation yields closed-form full-conditionals, which will become useful for the computational efficient inference algorithm introduced later. The linear predictor $x_{i}$ is derived from a *d*-dimensional vector $m_{i}={\left(m_{i1},\ldots ,m_{id}\right)}^{⊤}$ of observed features, i=1,…,n, and contains not only linear effects, but also interaction terms of the *d* features as illustrated in Fig. 1B. Our method is not limited to a specific effect type. Different types of interaction effects can be accommodated, guided by the structure of the application. For example, when considering an intercept, linear effects as well as all possible pairwise multiplicative interactions of the form $m_{il}m_{il^{\prime }}$, l=l′, the linear predictor $x_{i}^{⊤}\beta$ in (1) can be written as $\beta_{0}+\sum_{l=1}^{d}\beta_{l}m_{l}+\sum_{l=1}^{d}\sum_{l^{\prime }>l}\beta_{ll^{\prime }}m_{l}m_{l^{\prime }}$. Hence, *p* is typically much larger than *d*. This is the structure we consider in Section 4 when we apply BaGGLS to interaction discovery from motif scanner outputs. In this case $m_{i}$ will be a vector of attribution scores derived from a deep learning architecture. However, our general framework allows us to specify arbitrary effect and interaction terms including higher-order effects. It is important to note that first, even for small and moderate *d*, the number of potential interactions is large and thus $x_{i}$ can be high-dimensional. Second, we also explicitly allow for much larger *p* than the sample size *n*, that is, p≫n.

### 2.2 Overlapping group horseshoe prior

If the number of potential effect terms *p* is large, (1) can be challenging to interpret. Based on common scenarios in biological applications, we make the following assumptions. (i) We assume that β is sparse. That is, most entries are zero and thus only a small number of effect terms, $x_{ij}$, influence $y_{i}$. (ii) We further assume, that only a small subset of features $m_{il}$ significantly influences *y* and thus for most features all terms involving that feature are jointly zero.

The first assumption can be incorporated by considering an appropriate shrinkage prior. The prior structure also acts as an important regularization to the high-dimensional regression model. Here, we build on the popular horseshoe prior (Carvalho *et al*. 2010). The horseshoe prior introduces a global shrinkage parameter τ controlling the overall level of shrinkage jointly for all predictors and local shrinkage parameters $\lambda_{j}$  j=1,…,p controlling the shrinkage for the coefficient $\beta_{j}$ corresponding to effect $x_{ij}$. Xu *et al*. (2017) extend this prior structure to account for group-based shrinkage. The heredity assumption (ii) implies a structure with many overlapping groups. This would necessitate a prohibitively large number of hierarchies in the group horseshoe prior by Xu *et al*. (2017). We propose the following alternation to their grouped global-local shrinkage prior circumventing the need for many hierarchies for each predictor.

Let $J\in R^{p\times d}$ be an indicator matrix where entry $J_{jl}$ indicates if feature $m_{l}$ contributes to term $x_{j}$. We propose the hierarchical prior


$$\begin{array}{cc} \beta_{j}|\tau ,\lambda ,\delta \;∼\;ND\left(0,\tau \lambda_{j}\prod_{l=1}^{d}\delta_{l}^{J_{jl}}\right), & j=1,\ldots ,p\\ \tau |\nu \;∼IG\left(\frac{1}{2},\frac{1}{\nu }\right),\;\nu ∼IG\left(\frac{1}{2},1\right), & \\ \lambda_{j}|c_{j}\;∼IG\left(\frac{1}{2},\frac{1}{c_{j}}\right),\;c_{j}∼IG\left(\frac{1}{2},1\right), & j=1,\ldots ,p\\ \delta_{l}|t_{l}\;∼IG\left(\frac{1}{2},\frac{1}{t_{l}}\right),\;t_{l}∼IG\left(\frac{1}{2},1\right), & l=1,\ldots ,d, \end{array}$$

Where $\delta ={\left(\delta_{1},\ldots ,\delta_{d}\right)}^{⊤}$, $\lambda ={\left(\lambda_{1},\ldots ,\lambda_{p}\right)}^{⊤}$. Here, IG(α,β) denotes an inverse Gamma distribution with shape parameter α and scale parameter β. As for the standard horseshoe prior τ controls global shrinkage, and $\lambda_{j}$ controls local shrinkage. In addition, $\delta_{l}$ controls joint group shrinkage for all effects including feature $m_{l}$. The structure of the indicator matrix *J* informing the grouping structure depends on the effects considered in *x* and needs to be specified upfront. To allow for consistent interpretation of the effect strength as well as consistent shrinkage through the shared shrinkage parameters we assume that all effects $x_{ij},j=1,\ldots ,p$, are standardized to have unit scale across observations, i=1,…,n. A schematic description of our prior is given in Fig. 1C. Each term has an individual local shrinkage parameter $\lambda_{j}$. The motif dependent group shrinkage parameter $\delta_{l}$ is active exactly for the terms involving the corresponding feature $m_{l}$. The parameter τ controls global shrinkage and is shared across all terms. By construction of the prior, a great advantage of BaGGLS is that no upfront hyperparameter tuning is required. Only the effects included in the regression design, $x_{i}$, need to be specified. The full vector of the n+3p+2d+2 unknown model parameters is $\theta ={\left(z^{⊤},\beta^{⊤},\tau ,\nu ,\lambda^{⊤},c^{⊤},\delta^{⊤},t^{⊤}\right)}^{⊤}$.

### 2.3 Inference for large data sets

Exact Bayesian inference in the resulting high-dimensional probit model can be computationally challenging. VI emerged as a powerful alternative. The main idea illustrated in Fig. 1D is to learn an approximation to the posterior density p(θ∣y) using an approximating family of densities Q. Most commonly, the optimal approximation $q^{*}(\theta )$ is chosen so that it minimizes the reverse Kullback-Leibler divergence, $D_{\text{KL}}\left[q(\theta )||p(\theta |y))\right]=E_{q}\left[\text{log}\;q(\theta )-\text{log}\;p(\theta |y)\right],$ where $E_{q}[\cdot ]$ denotes expectation with respect to q(θ), among all approximating families in Q. A popular choice in logistic and probit regression models is the mean field assumption (e.g. Durante *et al*. 2019), which assumes independence between specific blocks of θ. This choice is computational efficient, but as recently shown by Fasano *et al*. (2022) can be too restrictive for large *p*. Therefore, the authors propose to relax the independence assumption between β and *z*. This allows for more flexibility in the posterior approximation, as it yields a unified skew–normal (Arrelano-Valle *et al*. 2006) approximation for the vector of regression coefficients. Fasano *et al*. (2022) consider a simple Gaussian prior for β. We extend their approach to the Gaussian scale mixture representation of the BaGGLS prior and consider variational approximations of the form


$$\begin{array}{cc} Q=\{q(\theta ):q(\theta ) & =q(\beta |z)\left(\prod_{i=1}^{n}q(z_{i})\right)q(\tau )q(\nu )\left(\prod_{j=1}^{p}q(\lambda_{j})q(c_{j})\right)\\ & \cdot \left(\prod_{l=1}^{d}q(\delta_{l})q(t_{l})\right)\}. \end{array}$$

We describe how $q^{*}(\theta )=\arg\min_{q(\theta )\in Q}D_{\text{KL}}\left[q(\theta )||p(\theta |y))\right]$ can be derived in a computational attractive manner using a simple coordinate ascent algorithm. The final algorithm updates one variational factor at a time while holding the others fixed, cycling through coordinates until convergence. Under the variational family Q given above all updates are in closed-form (see Appendix A, available as supplementary data at *Bioinformatics* online). It is possible to efficiently sample from q(β)=∫q(β∣z)q(z)dz even when p≫n using the strategies outlined in Bhattacharya *et al*. (2016) and the posterior mean $\hat{\beta }=E_{q}[\beta ]$ is given in closed form. Due to the variational approximation, samples from $q^{*}(\theta )$ will not yield exact uncertainty quantification, and Bayesian credible intervals for the regression coefficients may not achieve nominal coverage. It is thus not theoretically justified to test significance by checking whether zero lies outside a 95% credible interval. We propose to rely solely on the point estimator $\hat{\beta }$ for out of sample prediction and interpretation of the regression model. The high degree of sparsity imposed by BaGGLS makes $\hat{\beta }$ interpretable without requiring exact effect selection.

### 2.4 BaGGLS workflow

Figure 1 schematically outlines the BaGGLS workflow. Our method requires, a predefined p-dimensional vector of effects $x_{i}$ for observations i=1,…,n as inputs. While the method is general, we illustrate its use for post-hoc interpretation with Integrated Gradients (Sundararajan *et al*. 2017) applied on a genomic CNN with a binary classification. This is a common challenge in biological applications, where deep learning offers high predictive sharpness but limited interpretability, and this is also the setting of the application in Section 4. First, observation-specific features $m_{i}$, i=1,…,n, are selected, for example using attribution scores from a CNN aggregated within FIMO motif matches. Based on these features, a list of interpretable interactions needs to be defined. In our application, we include an intercept, linear effects, and all possible multiplicative pairwise interactions. Under this specification, BaGGLS can capture multiplicative interactions as well as contributions by individual features. Which interactions to include depends critically on the application and the goal of the analysis. We recommend to build on domain knowledge and avoid excessive redundancy or multi-colinearity to ensure identifiability. These choices fully define the prior and the design matrix. BaGGLS then returns a sparse vector of regression coefficients $\hat{\beta }$, resulting in a clear interpretation of the most important interaction terms. We suggest ranking the *p* terms by $\left|\beta_{j}\right|$, j=1,…,p as a simple approach to identify the top-*k* effects. This way complex interactions within attributions become interpretable. However, as a post-hoc approach BaGGLS cannot recover dependencies not learned by the upstream deep learning model.

## 3 Simulations

### 3.1 Illustrative example

#### 3.1.1 Data generating process

We generate m=500 data sets with n=500 independent observations. For $m_{i}=(m_{i1},\ldots ,m_{id})$ with $m_{ij}∼G(1,1)$, similar as in our application presented in Section 4 the corresponding vector of predictors $x_{i}$ consists of an intercept, linear effects $m_{j}$, j=1,…,d, and all possible pairwise interactions $m_{j}m_{j^{\prime }}$ for $j<j^{\prime }$. We set d=10, so that p=56 in our first set-up. After standardization of the design matrix, we generate observations $y_{i}∼\mathrm{Bern}(\Phi (x_{i}^{⊤}\beta^{*}))$, where all entries of $\beta^{*}$ are zero except for the entries corresponding to $m_{1}$, $m_{2}$, and the interaction $m_{1}m_{2}$. The true regression coefficient $\beta^{*}$ is thus extremely sparse and reflects the sparsity assumption described in Section 2.2. This data generating process allows for straight forward benchmarking with alternative penalization in probit regression. Detecting the active interaction can be challenging if n/p is small, and we vary the number of observations *n* and the number of observed features *d* in Section 3.2.

#### 3.1.2 Benchmark methods

We consider different regularization techniques for the probit model (1) that result in sparse and interpretable models. We use the same design matrix for all benchmarks, so that the model fits are directly comparable. In particular, we compare BaGGLS with:

UC: Unconstrained frequentist probit regression fitted via maximum likelihood,
L1: Frequentist probit regression with $L_{1}$-penalty and default hyperparameters as implemented in statsmodels (Seabold and Perktold 2010),
HS: MCMC sampling for the probit model equipped with the horseshoe prior (Carvalho *et al*. 2010) as implemented in brms (Bürkner 2017).

#### 3.1.3 Results

**Figure 2:**
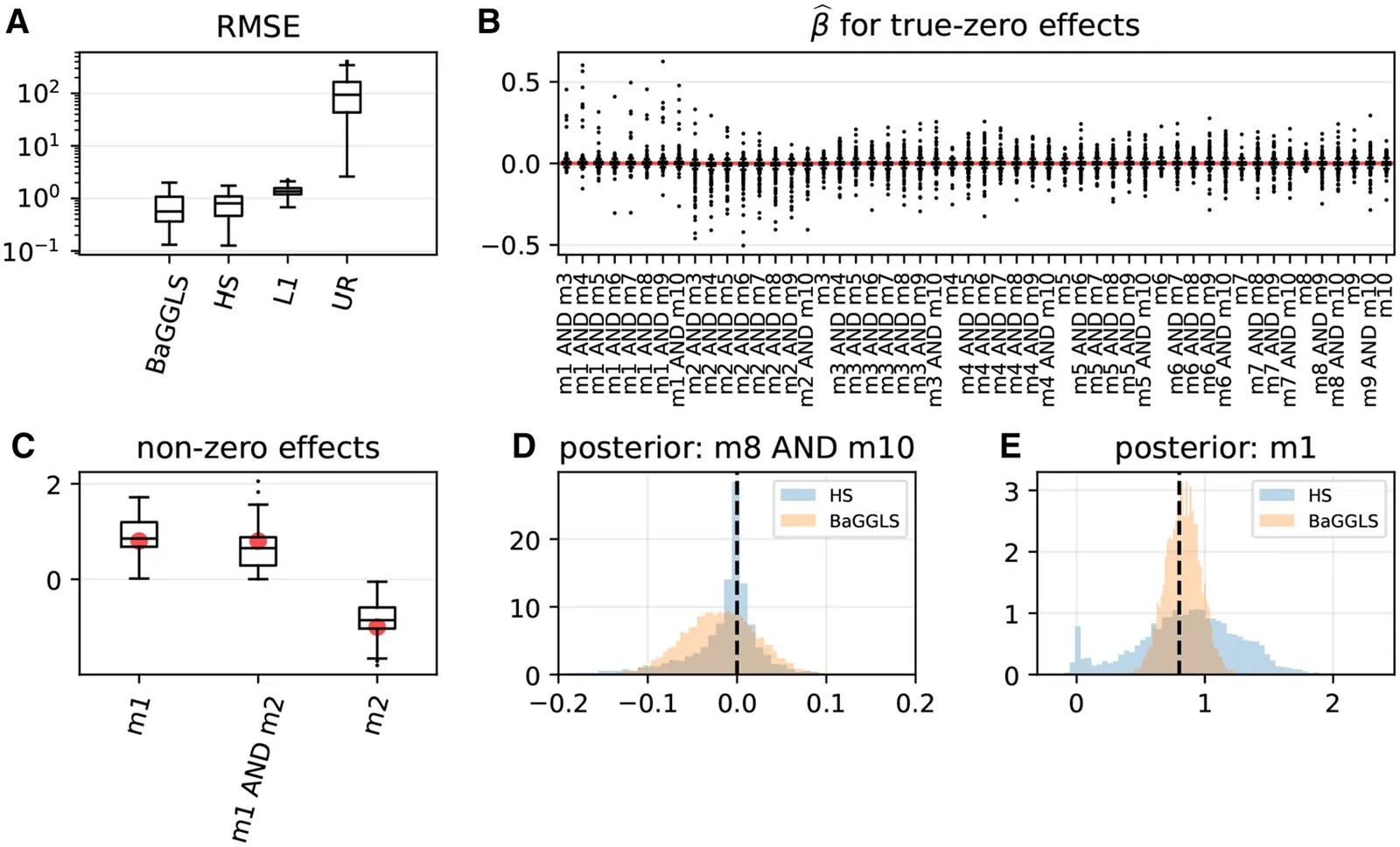
Simulations n=500,d=10. (A) RMSE across 500 independent runs on log-scale. Lower values are prefered. Our propossed method outperforms the benchmarks in recovery of the true coefficient vectors (B) Boxplots for ${\hat{\beta }}_{j}$ for all true-zero coefficients across 500 independent runs. For most repetitions, BaGGLS shrinks effects that do not contribute to the response successfully towards zero resulting in an interpretable model. (C) Boxplots for ${\hat{\beta }}_{j}$ for all non-zero coefficients. True values are marked by dots. BaGGLS correctly estimates the effect size for the three active effects in the data generating process. (D) Samples from the estimated marginal posterior for one true-zero coefficient for HS and BaGGLS. Both posteriors are correctly centered around zero and skewed indicating that the skewed variational approximation used for BaGGLS is helpful in tightly approximating the true posterior. (E) Samples from the estimated marginal posterior for one non-zero effect for HS and BaGGLS. Both posteriors are centered correctly. The variational approximation for BaGGLS is sharper than the posterior under HS.

Figure 2A shows boxplots of the root mean squared error (RMSE), $\text{RMSE}(\hat{\beta })={\left(\sum_{j=1}^{p}{\left(\beta_{j}^{*}-{\hat{\beta }}_{j}\right)}^{2}\right)}^{1/2}$, over the 500 independent repetitions. BaGGLS has the smallest average RMSE (0.7022), followed by HS (0.8052), and L1 (1.3827). Figure 2B plots estimates ${\hat{\beta }}_{j}$ for all coefficients with $\beta_{j}^{*}=0$ showing effective shrinkage toward zero. As expected, the largest variability occurs for joint effects involving $m_{1}$ or $m_{2}$. Non-zero effects (βj∗=0) are are accurately recovered (Fig. 2C). Figure 2D shows a histogram for a representative non-informative coeffiencent under BaGGLS and under HS from their respective marginal posteriors. Both are centered near zero and on a similar scale. Notably, the marginal posterior $q(\beta_{j})$ under BaGGLS is skewed, which is captured due to the unified skew-normal variational family. However, this family cannot capture the characteristic spike around zero. For a representative non-zero coefficient (Fig. 2E), BaGGLS concentrates more tightly around the truth, while HS posteriors are more dispersed. Nevertheless, both methods yield similar posterior means. Due to the variational approximation, posterior samples from BaGGLS will not yield exact uncertainty quantification. On this data, BaGGLS takes on average only 0.59 seconds and is therefore more than 100 times faster than MCMC sampling under the horseshoe prior rendering it suitable for large scale applications.

### 3.2 Scalability

#### 3.2.1 Data generating process

To further investigate the performance of BaGGLS we now vary the number of observations *n* and the number of observed features *d* in the data generative process described in Section 3.1. Due to the non-linear relationship between *d* and the number of effects *p* a slight increase in *d* leads to a large increase in *p* and thus to a much more challenging inference task. Here, we consider n=500 and n=2,000 as well as d=10,15,20 resulting in a total of 6 scenarios. These values are chosen to reflect common scenarios in real world data and result in a small sample-to-feature ratio n/p. This matches the general structure of the genomic data considered in Section 4.

#### 3.2.2 Performance metrics

In addition to the overall RMSE, we consider several other performance metrics. First, we evaluate discrimination via the area under the receiver operating characteristic curve (AUC) and probabilistic accuracy via the Brier score, $n^{-1}\sum_{i=1}^{n}(\Phi (x_{i}^{⊤}\hat{\beta })-y_{i}{)}^{2}$, both evaluated on an additional hold-out test data set with *n* = 10 000 observations. The Brier score is a proper scoring rule (Gneiting and Raftery 2007). These metrics are useful in comparing the out-of-sample predictive power of the different methods. Although prediction is not the primary objective of BaGGLS, reasonable out-of-sample performance remains desirable. However, when prediction is the main goal rather than interpretability, methods with weaker structural assumptions may be more suitable. To quantify effect recovery in high dimensions, we report RMSE computed separately over the active (nonzero) and inactive (zero) entries of $\beta^{*}$. In addition, we report the proportion of runs in which the three truly active effects are ranked among the top 20 and, separately, among the top 3 estimated effects by absolute magnitude. This mirrors typical workflows that select a small set of high-impact features for interpretation of the high-dimensional regression model. Lastly, we report average computation times on a standard laptop for all benchmarks.

#### 3.2.3 Results

All metrics are reported in Appendix B, available as supplementary data at *Bioinformatics* online. BaGGLS is compatible with the benchmark methods in terms of out of sample prediction measured by AUC and the Brier score, while more effective at detecting the active terms. Across all simulation scenarios, BaGGLS achieves the lowest RMSE on the active (nonzero) entries of $\beta^{*}$ followed by HS. HS imposes stronger shrinkage on zero coefficients as measured by the RMSE on the inactive components, particularly in scenarios with *n* = 2000, where the sample-to-feature ratio n/p is larger than in scenarios with n=500. In the application considered in Section 4 the sample-to-feature ratio n/p=1.33 is small.

Table 1 reports the proportion of repetitions in which the three truly active terms are ranked among the top 20 and, separately, among the top 3 estimated effects by absolute magnitude. While L1 and UR do not recover the active effects reliably, HS and BaGGLS place all three truly active effects in the top 20 for every run across all scenarios. However, BaGGLS outperforms HS when considering only the top 3 terms. In particular, the interaction effect $m_{1}m_{2}$ is often missed by HS. for n=500,d=20, the intercation appears in the top 3 in 88 of 100 repetitions for HS versus 93 of 100 for BaGGLS. Similarly, for n=500,d=15, BaGGLS detects the interaction 93 times, while HS places it outside the top 3 in 16% of runs. These results indicate that BaGGLS is robust at detecting interaction effects, especially when the sample-to-feature ratio is small. In addition, BaGGLS is between 7 and 122 times faster than HS.

**Table 1:** Simulations. Proportions of runs for which the truly active terms $m_{1}$, $m_{2}$, and $m_{1}m_{2}$ are among the top 20 or top 3 terms respectively for each benchmark method and all simulation scenarios.

	% in top 20 terms			% in top 3 terms			time (in s)
	$m_{1}$	$m_{2}$	$m_{1}m_{2}$	$m_{1}$	$m_{2}$	$m_{1}m_{2}$	
*n = 500, d = 10, p = 56, n/p = 8.93*							
BaGGLS	100%	100%	100%	94%	96%	94%	0.5866
HS	100%	100%	100%	91%	99%	91%	71.5908
L1	81%	91%	81%	53%	75%	53%	0.3983
UR	98%	98%	98%	44%	40%	44%	0.0238
*n = 2000, d = 10, p = 56, n/p = 35.71*							
BaGGLS	100%	100%	100%	100%	100%	100%	10.2991
HS	100%	100%	100%	100%	100%	100%	250.9252
L1	94%	100%	94%	85%	99%	85	0.4775
UR	98%	99%	98%	46%	50%	46%	4.3269
*n = 500, d = 15, p = 121, n/p = 4.13*							
BaGGLS	100%	100%	100%	93%	96%	93%	7.8429
HS	100%	100%	100%	84%	98%	84%	90.8542
L1	49%	66%	49%	21%	44%	21%	6.7732
UR	95%	84%	95%	68%	31%	68%	5.9180
*n = 2000, d = 15, p = 121, n/p = 16.53*							
BaGGLS	100%	100%	100%	100%	100%	100%	26.7289
HS	100%	100%	100%	100%	100%	100%	298.2052
L1	88%	99%	88%	61%	92%	61%	5.7420
UR	92%	94%	92%	48%	49%	48%	6.8981
*n = 500, d = 20, p = 211, n/p = 2.37*							
BaGGLS	100%	100%	100%	93%	94%	93%	15.6204
HS	100%	100%	100%	88%	99%	88%	107.5822
L1	13%	19%	13%	8%	13%	8%	71.3119
UR	–	–	–	–	–	–	–
*n = 2000, d = 20, p = 211, n/p = 9.48*							
BaGGLS	100%	100%	100%	100%	100%	100%	48.2972
HS	100%	100%	100%	100%	100%	100%	371.5898
L1	71%	88%	71%	41%	72%	41%	34.3788
UR	75%	81%	75%	47%	49%	47%	12.4084

The last column reports the average run-time in seconds on a standard laptop. UR did not reliably converge for the scenario n=500, d=20. BaGGLS outperforms the benchmarks in detecting relevant effects including the interaction.

## 4 Application to genomic attribution scores

As a proof-of-concept, we illustrate how BaGGLS is useful as a post hoc processing method for deep learning explanations. To this end, we apply it to attribution scores derived from deep learning models trained on semi-synthetic genomic sequences containing real motifs and known ground truth. The use of real motifs ensures that the datasets are realistic while the known ground truth allows a direct evaluation, which would be impossible on real data.

### 4.1 Data and motif scanning

**Figure 3:**
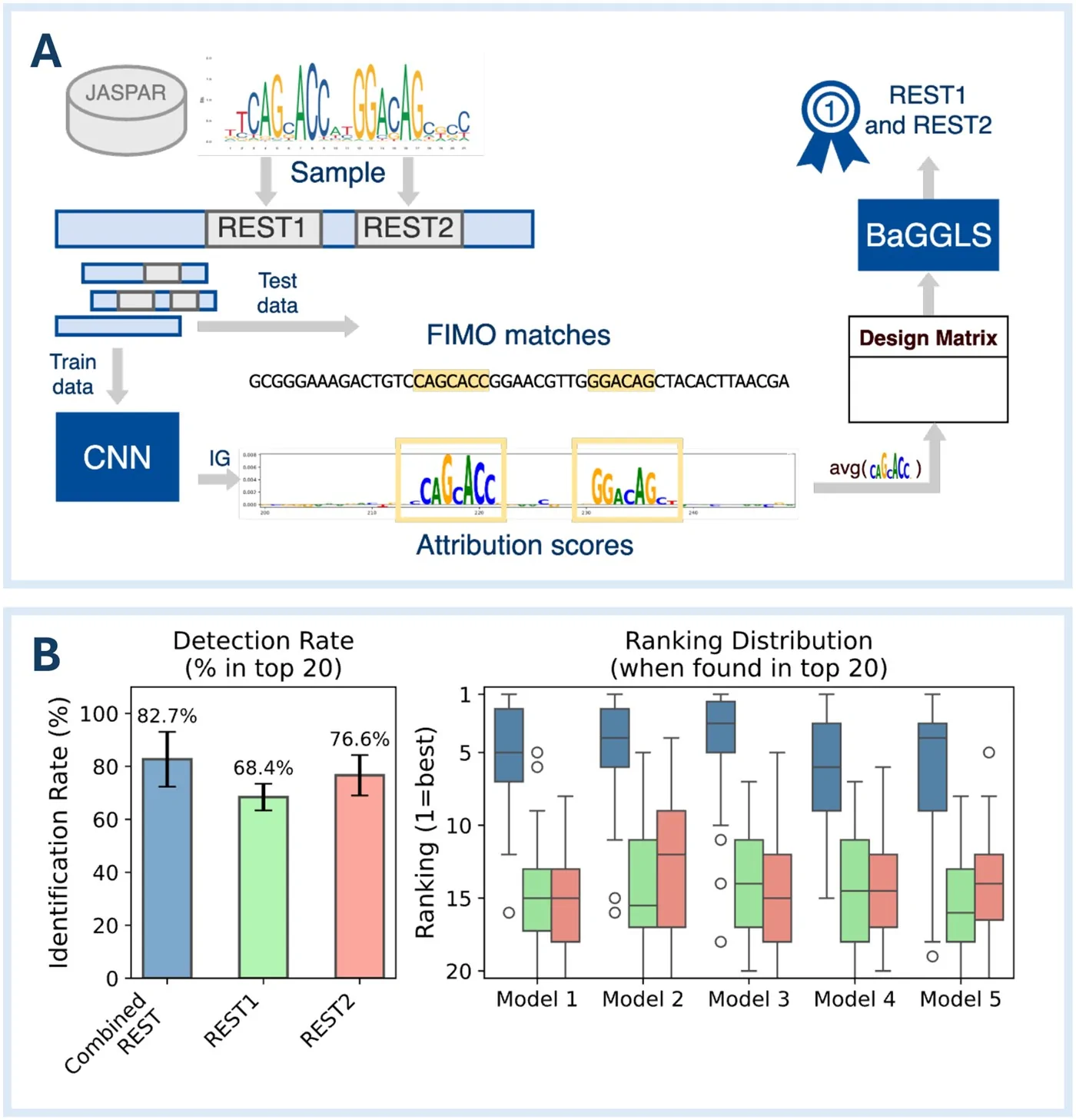
Post-processing of BaGGLS on attribution scores. (A) First, we generated synthetic sequences by inserting the sampled composite REST motif in 500 bp long random sequences. We trained five shallow CNN models on that data and interpreted the test data with Integrated Gradients. By applying FIMO on the sequences, we obtain matches which resemble motifs from the JASPAR2024 data base. We average the attribution scores in the matched motif region and compute the design matrix and indicator matrix from those scores for BaGGLS. (B) We applied BaGGLS on the 45 test data sets for each model and measured how often the interaction term was included in the Top 20 terms. BaGGLS ranks the interaction term on average in 82.7% of the test data sets as a top 20 effect while the individual effects in 68.4% and 76.6% for the first and second half of the REST motif respectively (left plot). When looking closer to the exact ranking of the identified terms (right plot), the interaction term receives much higher ranking than the individual effects.

The balanced dataset consists of synthetic DNA sequences with binary labels indicating the presence of a motif set of interest. We follow a similar simulation protocol as described in Tseng *et al.* (2024) to evaluate the approach on known ground truth. Depending on the defined motif grammar, the motifs are inserted in randomly generated sequence. Here, we explored the REST motif consisting of two submotifs with a specific order and spacing (Fig. 3A). 35 000 sequences are generated for training and 10 000 for validation, with a sequence length of 500 base pairs. To evaluate BaGGLS, we generated additional 45 evaluation datasets with the same grammar each consisting of 2000 sequences. FIMO locates motifs by computing significant matches of motifs with position-weight matrices (PWM) from databases in a sequence. Here, we use all latest versions of the human transcription binding site motifs (d=755) in JASPAR2024. Depending on the *P* value threshold, the results contain many false positive matches (high threshold) or miss some of the real motif matches (low threshold). We use the default threshold $\mathrm{pthresh}=1e^{-4}$ to investigate the robustness of BaGGLS by exposing it to noisy motif matches by allowing false positives as well as relevant motifs which are not matched by FIMO despite being present.

### 4.2 Attribution scores from deep learning models

We train five shallow CNNs on the training data set. We specifically use a shallow architecture to not overfit to the synthetic data resulting in an average AUC of 0.91. Details on the architecture and performance can be found in the supplement section C, available as supplementary data at *Bioinformatics* online. The trained CNNs are interpreted by the post-hoc attribution method Integrated Gradients. Integrated gradients measure how much each input feature contributes to the output by accumulating gradients along a path from a reference baseline to the actual input. For simplicity, we use a zero baseline. We calculate the scores for all evaluation data sets for each of the five CNNs.

### 4.3 Attribution-based design matrix

BaGGLS requires a pre-defined set of features and interactions passed in a design matrix (Fig. 1B). Here, we pass all matched motifs by FIMO as possible features to BaGGLS. FIMO returns the start and end positions of the matched motifs within each sequence. With that information, we can aggregate the attribution scores in that subregion to obtain motif-wise contributions. Here, we use the absolute average from the attribution scores at the matched motif positions scaled sequence-wise so that the aggregated motif scores add up to 1. To create interaction features, we calculate the pairwise co-activation based on the product of the average contribution scores. We remove rarely occurring features to avoid inefficiency due to a very large number of features by using a cut-off quantile of 0.95 which can be adjusted by the user. The resulting features still comprise of a large number of motif and interaction features (*P* = 1500), resulting in a ratio of n/p=1.33 similar to the scenarios considered in Section 3. The design matrix consists of 755 single effects and 745 interaction effects. Similarly as in the simulation, we fit BaGGLS for each of the 45 evaluation data sets for each of the 5 CNNs.

### 4.4 Attribution evaluation

Due to the sparsity assumption only a small sub-set of regression coefficients $\hat{\beta }$ derived by BaGGLS is meaningfully different from 0. This is the set of effectively active feature and interaction effects detected by BaGGLS. For further interpretation, we calculate the top-20 effects by absolute magnitude of their respective coefficients (see Fig. 1F). This can be viewed as the set of the most important 20 effects out of the *p* = 1500 potential ones passed to our method.

For illustration purposes, we consider here data with a known ground truth. This allows us to evaluate whether BaGGLS captures the known main interaction, by checking how often the interaction is included within the top-20 effects. BaGGLS identifies the interaction term in 82.7% of the scenarios whereas the REST submotifs were included within the top-20 in 68.4% and 76.6% of the scenarios, respectively (Fig. 3A). It is important to note that when using BaGGLS as post-processing method, its performance is highly dependent on the fit of the base CNN. In particular BaGGLS cannot detect effects that were overlooked by the base model. When analyzing at the rankings of the terms for each of the five CNN models individually (Fig. 3B), the interaction term ranks on average higher than the individual submotif effects for each model (Median ranks: interaction term = 4, REST1 = 15, REST2 = 14). This shows that BaGGLS robustly identifies the driving interaction among noisy attribution scores and prioritizes the interaction over individual effects. For this reason, BaGGLS can be used as highly interpretable post-hoc processing method for trained deep learning genomic classifiers. Identifying important interactions is not directly possible from the CNNs, but crucial for understanding the underlying biological process.

## 5 Discussion

BaGGLS is an interaction detection method for high-dimensional, noisy biological data. We make the following main contributions. (i) We propose to use a structured probit regression model as a post-hoc analysis tool for deep classifiers. Our model takes a large number of potential effect, including interactions, as input and identifies a small subset of important and truly active effects. (ii) Sparsity is imposed through a novel shrinkage prior that respects the overlapping group structure resulting from the inclusion of many interaction terms in the additive predictor and that does not require any cumbersome hyperparameter tuning. (iii) We propose a fast algorithm for posterior inference based on a unified skewed-normal approximation. This is crucial for scalability to scenarios with many potential effects and a a small sample-to-feature ratio. (iv) We conduct an extensive simulation study, and show that BaGGLS outperforms state-of-the-art benchmarks in detecting relevant interaction effects. (v) We illustrate the merits of our approach in an application on motif interactions in genomic data which is known to be complex and noisy offering a good use case for comparisons. While we rely on a specific setup of tools for the initial learning and post-hoc interpretation, BaGGLS is a generic method, not restricted to the specific setup chosen here.

Even though, our general framework allows for arbitrary and more complex effect types to be specified, we have only considered continuous features and interactions based on co-occurrences within our application. The inclusion of more complex effects, for example, by including binary features and boolean interactions in the context of logic regression (Ruczinski *et al*. 2003) coupled with regulatory logic in genomics (Buchler *et al*. 2003) is a promising direction.

Combining our novel overlapping group shrinkage prior with regression models beyond the probit model, is a further possibility. For example, current deep learning prediction tasks in the genomic domain shift from classification to continuous and categorical responses by considering biological signals like gene expression or read counts directly. Quantitative readouts provide a more fine-grained picture of a biological signal. Extending BaGGLS to continuous responses might be one way to account for low-affinity motifs which are sometimes ignored by peak calling methods (Zeitlinger 2020).

While we demonstrate application to motif detection, other biological domains can also benefit from BaGGLS. In genetics, single-nucleotide polymorphisms are studied in the context of disease. While rare disorders are frequently driven by individual variants with large effects, common-variant contributions to complex traits are highly polygenic and often involve context-dependent effects that are hard to resolve in high-dimensional genotype space (Wray *et al*. 2018). Currently, regulatory genomics is shifting from bulk assays to single-cell and related high-resolution assays. These provide finer cell-type specificity but produce sparser, noisier data matrices that change the statistical challenges and opportunities (Bouland *et al*. 2023). This shift opens new applications for BaGGLS.

However, there are also limitations. Model architecture and performance strongly affects model interpretability as well as motif representation. We have to assume that the model captures the biological mechanisms to some extent and leave the exploration of how interpretability of interactions depends on model architectures to other research (Thompson and Lehner 2025). Besides the logical relationship between motifs, spacing, orientation, and ordering of motifs influences the motifs’ effect on the a signal. In some genomic regions, composite regulatory elements, which are arrangements of multiple nearby binding sites, can alter local sequence preferences and functional readout (Jolma *et al*. 2015). Bulk models that assume independent binding sites often miss these dependencies, which leads to only a sparse but important set of interactions being captured. Adjusting the design matrix to include interaction terms based on experimentally validated composite elements can therefore make interaction interpretations more precise. This can further be improved by an iterative approach in which interpretable features from deep models, for example, convolutional filters (Tseng *et al.* 2024) or in-silico perturbation readouts (Gjoni *et al.* 2024), are used to detect candidate composite arrangements that are then incorporated into the design matrix to refine sparsity structure and interaction estimates. The interaction patterns detected by our method could also serve as hypotheses for previously unrecognized composite elements or motif interactions. These hypotheses can be ranked by BaGGLS and subsequently tested in targeted biochemical or reporter assays, providing a systematic way to identify novel regulatory interactions.

## Supplementary Material

btag441_Supplementary_Data

## Data Availability

The simulated and exemplary data underlying this article are available in the repository or at https://doi.org/10.5281/zenodo.17675631.
