# Supplementary material for: BaGGLS: a Bayesian shrinkage framework for interpretable modeling of interactions in high-dimensional biological data
Source: Bioinformatics. 2026 Aug 21;42(Suppl 2):btag441. doi: 10.1093/bioinformatics/btag441 (PMC13501314; doi:10.1093/bioinformatics/btag441)
Supplement: Supplementary file 1 [file btag441_supplementary_data.pdf]

A. Variational Inference

The full vector of the $n+3p+2d+2$ unknown model parameters is $\theta = (z^\top, \beta^\top, \tau, \nu, \lambda^\top, c^\top, \delta^\top, t^\top)$. We consider the following variational approximation

$$q(\theta) = q(\beta \mid z) \left(\prod_{i=1}^{n} q(z_i) \right) q(\tau) q(\nu) \left(\prod_{j=1}^{p} q(\lambda_j) q(c_j) \right) \left(\prod_{l=1}^{d} q(\delta_l) q(t_l) \right). \tag{2}$$

Note that (2) is not the fully factorized mean-field approximation as we do neither assume independence between β and z nor between the individual entries of β. Instead we consider the partially factorized approximation introduced by A. Fasano et al. (2022). The structure (2) implies that $q^*(\beta \mid z)$ is a p-dimensional Gaussian distribution, $q^*(z_i)$ is a truncated normal distribution and all remaining factors of $q^*(\theta)$ are inverse gamma distributions. We can thus write

$$\begin{aligned}
q^*(\beta \mid z) &= \phi_p(\beta; B(\beta) z, \Sigma(\beta)); \\
q^*(z_i) &= 1_{(2y_i-1)z_i>0} \frac{\phi(z_i; \mu(z_i), \sigma^2(z_i))}{\Phi((2y_i-1)\mu(z_i)(\sigma^2(z_i))^{-\frac{1}{2}})} && i = 1, \dots, n; \\
q^*(\tau) &= p_{\mathcal{IG}}(\tau; a(\tau), b(\tau)); \\
q^*(\nu) &= p_{\mathcal{IG}}(\nu; a(\nu), b(\nu)); \\
q^*(\lambda_j) &= p_{\mathcal{IG}}(\lambda_j; a(\lambda_j), b(\lambda_j)) && j = 1, \dots, p; \\
q^*(c_j) &= p_{\mathcal{IG}}(c_j; a(c_j), b(c_j)) && j = 1, \dots, p; \\
q^*(\delta_l) &= p_{\mathcal{IG}}(\delta_l; a(\delta_l), b(\delta_l)) && l = 1, \dots, d; \\
q^*(t_l) &= p_{\mathcal{IG}}(t_l; a(t_l), b(t_l)) && l = 1, \dots, d.
\end{aligned}$$

The optimal variational parameters are given as

$$\begin{aligned}
\Sigma(\beta) &= \left(X^\top X + \operatorname{diag}\left(\frac{a(\tau)a(\lambda_1)}{b(\tau)b(\lambda_1)} \prod_{J_{1l}=1} \frac{a(\delta_l)}{b(\delta_l)}, \dots, \frac{a(\tau)a(\lambda_p)}{b(\tau)b(\lambda_p)} \prod_{J_{pl}=1} \frac{a(\delta_l)}{b(\delta_l)} \right) \right)^{-1} \\
B(\beta) &= \Sigma(\beta) X^\top \\
\mu(z_i) &= \sigma^2(z_i) x_i^\top \Sigma(\beta) X_{-i}^\top (\mathbb{E}_q[z_1], \dots, \mathbb{E}_q[z_{i-1}], \mathbb{E}_q[z_{i+1}], \dots, \mathbb{E}_q[z_n])^\top \\
\sigma^2(z_i) &= \left(1 - x_i^\top \Sigma(\beta) x_i\right)^{-1} \\
a(\tau) &= \frac{p+1}{2} \\
b(\tau) &= \frac{1}{2} \sum_{j=1}^{p} \left(\mathbb{E}_q[\beta_j^2] \frac{a(\lambda_j)}{b(\lambda_j)} \prod_{J_{jl}=1} \frac{a(\delta_l)}{b(\delta_l)} \right) + \frac{a(\nu)}{b(\nu)} \\
a(\nu) &= 1 \\
b(\nu) &= \frac{a(\tau)}{b(\tau)} + 1 \\
a(\lambda_j) &= 1 \\
b(\lambda_j) &= \frac{1}{2} \mathbb{E}_q[\beta_j^2] \frac{a(\tau)}{b(\tau)} \prod_{\mathbf{J}_{jl}=1} \frac{a(\delta_l)}{b(\delta_l)} + \frac{a(c_j)}{b(c_j)} \\
a(c_j) &= 1 \\
b(c_j) &= \frac{a(\lambda_j)}{b(\lambda_j)} + 1 \\
a(\delta_l) &= \frac{\sum_{j=1}^{p} J_{jl} + 1}{2} \\
b(\delta_l) &= \frac{a(\tau)}{b(\tau)} \sum_{j=1}^{p} J_{jl} \frac{a(\lambda_l)}{b(\lambda_l)} \mathbb{E}_q[\beta_j^2] + \frac{a(t_l)}{b(t_l)} \\
a(t_l) &= 1 \\
b(t_l) &= 1 + \frac{a(\delta_l)}{b(\delta_l)},
\end{aligned}$$

where

$$\begin{aligned}
\mathbf{E}_q[\beta_j^2] =& (\Sigma(\beta) + \Sigma(\beta) X^\top \\
&\operatorname{diag}(\sigma^2(z_1) - (\mathbf{E}[z_1] - \mu(z_1))\mathbf{E}[z_1], \dots \\
&, \sigma^2(z_n) - (\mathbf{E}[z_n] - \mu(z_n))\mathbf{E}[z_n]) X \Sigma(\beta))_{jj} \\
&+ (B(\beta)(\mathbf{E}[z_1], \dots, \mathbf{E}[z_n])^\top)_j^2; \\
\mathbf{E}_q[z_i] =& \mu(z_i) + (2y_i - 1)\sqrt{\sigma^2(z_i)} \frac{\phi\left(\mu(z_i)\sigma^2(z_i)^{-\frac{1}{2}}\right)}{\Phi\left((2y_i-1)\mu(z_i)\sigma^2(z_i)^{-\frac{1}{2}}\right)}.
\end{aligned}$$

We refer to A. Fasano et al. (2022) for technical details in the derivation of $q^*(\beta, z)$. Using this set of equations $q^*(\theta)$ can be updated via coordinate ascent (Bishop, 2006). Note that each step involves inverting the $p \times p$ dimensional matrix $\Sigma(\beta)$. If $p > n$, the Woodbury matrix identity is used to invert a lower dimensional $n \times n$ matrix instead.

B. Additional simulation results

Table 2 summarizes the overall RMSE, the RMSE computed separately on the active (nonzero) and inactive (zero) entries of β^*, the area under the receiver operating characteristic curve (AUC), and the Brier score, $n^{-1} \sum_{i=1}^{n} (\Phi(x_i^\top \widehat{\beta}) - y_i)^2$, evaluated on an additional hold-out test data set with $n = 10{,}000$ observations. We report average values as well as standard deviations (in brackets) over 100 repetitions.

C. Application to genomic attribution scores

This section gives additional details on the deep learning models considered within our application presented in Section 4 of the main paper. The architecture first employs a one-dimensional convolutional layer with 8 filters (kernel size = 8), designed to scan the input sequences for motif patterns. The output of this layer is processed by a ReLU activation function and batch normalization. This is followed by two fully connected layers, each with 16 units and ReLU activations. To mitigate overfitting, a dropout layer with a rate of 0.3 is applied after each fully connected layer. The architecture concludes with a dense output layer followed by a sigmoid activation function, which outputs a probability score representing the likelihood of the input sequence containing the target motif set. We trained 5 models and evaluated the predictive performance on a separate test data set consisting of 2000 samples (see Table 3).

	RMSE (↓)	RMSE non zero (↓)	RMSE true-zero (↓)	AUC (↑)	Brier score (↓)
n=500, d=10, p=56, n/p=8.93					
BaGGLS	**0.7022** (0.4180)	**0.5627** (0.3889)	**0.3881** (0.2220)	0.8648 (0.0040)	0.1483 (0.0026)
HS	0.8052 (0.3671)	0.6876 (0.3388)	**0.3964** (0.1960)	**0.8660** (0.0031)	**0.1478** (0.0020)
L1	1.3827 (0.2706)	0.6892 (0.2880)	1.1727 (0.2278)	0.8457 (0.0063)	0.1616 (0.0042)
UR	114.2356 (85.8997)	14.4545 (10.4597)	113.2603 (85.3364)	0.7569 (0.0845)	0.2601 (0.0839)
n=2000, d=10, p=56, n/p=35.71					
BaGGLS	**0.2356** (0.1305)	**0.1775** (0.1159)	**0.1381** (0.0924)	0.8674 (0.0013)	0.1472 (0.0007)
HS	**0.2186** (0.1247)	**0.1737** (0.1196)	**0.1191** (0.0683)	**0.8676** (0.0010)	**0.1471** (0.0006)
L1	0.7699 (0.1804)	0.4266 (0.2162)	0.6206 (0.1074)	0.8611 (0.0019)	0.1510 (0.0012)
UR	59.8707 (44.3895)	7.3706 (5.4846)	59.4063 (44.0615)	0.8398 (0.0245)	0.1736 (0.0245)
n=500, d=15, p=121, n/p=4.13					
BaGGLS	**0.8325** (0.4839)	**0.6967** (0.4367)	**0.4219** (0.2704)	0.8533 (0.0058)	**0.1558** (0.0036)
HS	**0.9311** (0.3800)	0.8220 (0.3459)	**0.4177** (0.2040)	**0.8558** (0.0035)	**0.1545** (0.0023)
L1	2.3274 (0.3461)	0.8842 (0.3459)	2.1265 (0.3364)	0.8056 (0.0100)	0.1956 (0.0073)
UR	86.4223 (67.9976)	11.6303 (7.5754)	85.0680 (68.2881)	0.7597 (0.0383)	0.2590 (0.0384)
n=2000, d=15, p=121, n/p=16.53					
BaGGLS	0.2267 (0.1152)	**0.1673** (0.1031)	0.1410 (0.0787)	0.8614 (0.0012)	0.1504 (0.0007)
HS	**0.1994** (0.1115)	**0.1653** (0.1063)	**0.1029** (0.0546)	**0.8621** (0.0007)	**0.1500** (0.0004)
L1	1.2030 (0.2149)	0.5456 (0.2493)	1.0519 (0.1639)	0.8459 (0.0029)	0.1603 (0.0018)
UR	53.0565 (33.0476)	4.9558 (2.7377)	52.8008 (32.9721)	0.8287 (0.0162)	0.1788 (0.0174)
n=500, d=20, p=211, n/p=2.37					
BaGGLS	**0.8575** (0.4544)	**0.7135** (0.4310)	0.4288 (0.2511)	0.8476 (0.0076)	0.1593 (0.0044)
HS	**0.9166** (0.3401)	0.8283 (0.3253)	**0.3707** (0.1627)	**0.8516** (0.0035)	**0.1571** (0.0021)
L1	3.9889 (0.5015)	1.3117 (0.3719)	3.7540 (0.4599)	0.7648 (0.0114)	0.2447 (0.0091)
UR	–	–	–	–	–
n=2000, d=20, p=211, n/p=9.48					
BaGGLS	0.2388 (0.1003)	**0.1709** (0.0942)	0.1528 (0.0754)	0.8565 (0.0016)	0.1536 (0.0009)
HS	**0.2050** (0.0984)	**0.1710** (0.0945)	**0.1045** (0.0513)	**0.8576** (0.0007)	**0.1529** (0.0005)
L1	1.7003 (0.2597)	0.7289 (0.2766)	1.5154 (0.2332)	0.8294 (0.0038)	0.1724 (0.0027)
UR	37.2856 (24.8385)	3.4586 (2.0772)	37.0293 (24.8942)	0.8184 (0.0096)	0.1826 (0.0080)

Table 2 Simulations. Average values and standard deviations (in brackets) across all independent repetitions for the six simulation scenarios for the overall RMSE, the RMSE computed on the active (nonzero) and inactive (zero) entries of β^*, the area under the receiver operating characteristic curve (AUC), and the Brier score. Bold values indicate the best performing method in terms of the best mean value as well as methods potentially tied according to a one-sided t-test with level $\alpha = 0.05$. UR did not reliably converge for the scenario $n = 500, d = 20$.

Model:	Model 1	Model 2	Model 3	Model 4	Model 5
AUC	0.9157 ± 0.0074	0.9137 ± 0.0060	0.9037 ± 0.0069	0.9088 ± 0.0062	0.9099 ± 0.0067

Table 3 Performance of the CNNs on the 45 evaluation sequence datasets with each 2000 sequences. AUC are calculated on each dataset separately, since they were used separately for fitting BaGGLS. On average, an AUC of 0.91 could be achieved.
